# Plant Trait-Based Life Strategies of Overlapping Species Differ Between Anthropogenically Disturbed and Conserved Forests

**DOI:** 10.3390/plants15192962

**Published:** 2026-09-28

**Authors:** Haojun Xia, Chenchen Cai, Jianwei Liao, Kaiyuan Li, Libin Liu

**Affiliations:** 1 College of Life Sciences, Zhejiang Normal University, Jinhua 321004, China; haojun.xia@outlook.com; 2 The Administration Center of Zhejiang Jiulong Mountain National Nature Reserve, Lishui 323312, China; jiulongshanccc@163.com (C.C.); scxljw@163.com (J.L.); wldtksky86@163.com (K.L.); 3 College of Life Sciences, Zhejiang University, Hangzhou 310058, China

**Keywords:** morphological traits, ecological stoichiometry traits, anthropogenic disturbances, environments, adaptive strategy, subtropical forests

## Abstract

Global forest ecosystems face escalating threats from intensive anthropogenic disturbances. Plants inhabiting forests with contrasting disturbance histories often evolve divergent life history strategies, as reflected in functional trait differences across heterogeneous environments. However, most previous studies have inadequately controlled for the confounding effect of species identity, as anthropogenic disturbances markedly alter community composition; such compositional turnover obscures the precise attribution of trait variation to environmental drivers. In this study, we comprehensively assessed 11 morphological traits and 24 ecological stoichiometry traits across 23 overlapping plant species distributed in both anthropogenically disturbed and well-conserved subtropical forests in eastern China. We systematically evaluated trait characteristics and their implications for life history strategies in disturbed versus conserved forests. Our results revealed that plants in disturbed forests exhibited significantly greater leaf thickness, higher dry matter content and tissue density in twigs, bark, and stems, and elevated leaf dry matter content, accompanied by reduced leaf area and specific leaf area, as well as lower nitrogen concentrations in leaves and twigs and lower phosphorus concentrations in bark and stems. Collectively, these traits reflect a resource-conservative strategy. In contrast, plants in conserved forests displayed opposing trait syndromes, indicative of a resource-acquisitive strategy. Notably, when analyses were restricted to overlapping species, no evidence of reduced functional trait variability was observed in disturbed forests. This study elucidates how anthropogenic disturbances drive divergent plant functional adaptations and enhances regional and global trait databases.

## 1. Introduction

Forests constitute cornerstone components of terrestrial ecosystems and perform indispensable roles in sustaining global biodiversity, mitigating climate change, and conserving water and soil resources [1,2,3]. Globally, forests are increasingly subjected to anthropogenic disturbances, including timber harvesting, livestock grazing, conversion to agricultural land, tourism-related activities, and infrastructure development, leading to homogenization of species composition, structural fragmentation of communities, and progressive deterioration of ecosystem functioning in anthropogenically disturbed forests [4,5,6]. Consequently, anthropogenic disturbances have become a principal driver influencing forest species regeneration, community assembly, and ecosystem stability and dynamics [7,8,9,10,11]. A systematic understanding of the ecological consequences of such disturbances and of the adaptive strategies employed by plants inhabiting these altered environments is essential for the targeted conservation of primary forests, the effective restoration and rehabilitation of degraded forests, and the advancement of globally applicable, science-informed sustainable forest management practices [12,13,14].

Plant functional traits refer to morphological, physiological, and phenological characteristics that reflect how plants adapt to abiotic and biotic environmental conditions throughout their life cycles [15]. Individually or in combination, these traits encapsulate plant strategies for optimizing the acquisition, utilization, and allocation of resources, thereby serving as a critical functional interface linking plants, their environments, and ecosystem processes [16,17,18]. Traditional approaches centered on analyzing community species composition and its temporal dynamics to infer underlying ecological mechanisms are increasingly inadequate. In contrast, the growing emphasis on functional trait-based research enables ecologists to quantitatively assess and predict how disturbances influence biodiversity patterns [19,20]. In recent years, key functional traits, including specific leaf area (SLA), leaf dry matter content (LDMC), leaf nitrogen and phosphorus concentrations (LN and LP), and wood density, have been widely applied to quantify the extent and intensity of forest disturbance, identify plant adaptive strategies, yield novel insights into disturbance-driven evolution trajectories of forest ecosystems, and inform scientifically robust species selection and spatial design in forest restoration initiatives [21,22,23,24,25,26].

The uplift of the Qinghai–Tibetan Plateau profoundly reconfigured East Asian monsoon circulation, triggering the formation of an extensive subtropical zone across southern China. Within this zone, zonal subtropical evergreen broad-leaved forests constitute a globally unique biome—distinct from all other ecosystems at comparable latitudes [27,28]. These forests deliver irreplaceable ecological services, including exceptionally high biodiversity, substantial carbon sequestration capacity, and significant air and water pollutant purification [29,30,31]. Nevertheless, this subtropical region overlaps with China’s most economically dynamic and densely populated areas. Decades of intensive anthropogenic disturbances, such as clear-cut logging, understory cultivation, and habitat fragmentation, have severely diminished intact primary evergreen broad-leaved forests, leaving only isolated, small remnants of the original forest stands [32,33]. In recent decades, numerous studies conducted in this region—leveraging plant functional traits and trait-based life history strategies—have consistently demonstrated that plant communities in well-conserved forests exhibit markedly distinct trait characteristics and life history strategies compared with those in anthropogenically disturbed forests [4,25,34,35,36,37,38,39]. For instance, Guan et al. [25] documented pronounced differences in both morphological and ecological stoichiometry traits among dominant plant species between conserved and disturbed forests; they further reported reduced trait variability and a systematic shift toward a resource-conservative strategy in disturbed forests. Nevertheless, the vast majority of published studies have centered on interspecific comparisons of dominant species between two forest types, largely neglecting intraspecific trait variation and the ecological roles of species (commonly termed overlapping species) that co-occur across both conserved and anthropogenically disturbed habitats [4,38]. This critical knowledge gap hinders the ability to disentangle intraspecific trait variation from interspecific turnover, thereby constraining mechanistic understanding of how forest functional composition responds to anthropogenic disturbances. Consequently, targeted research investigating functional traits and trait-based life history strategies in overlapping species across disturbed and conserved subtropical forests is not only highly warranted but also urgently needed to advance trait-based community assembly theory.

In this study, we examined 23 dominant overlapping plant species that co-occur in anthropogenically disturbed and conserved forests in eastern China. A comprehensive suite of functional traits, including 11 morphological traits and 24 ecological stoichiometry traits, was quantified across four plant organs: leaves, twigs, bark, and stems. Functional trait characteristics and trait-based life history strategies were systematically compared between plants growing in the two forest types. Specifically, we tested the following two hypotheses: (1) plant functional traits and their intraspecific variation differ significantly between disturbed and conserved forests, and (2) disturbed forests exhibit reduced trait variability and distinct trait syndromes characterized by low SLA, nitrogen, and phosphorus concentrations across all four organs, coupled with high leaf thickness (LT), dry matter content, tissue density, and carbon concentration in the same organs—reflecting a resource-conservative strategy. In contrast, conserved forests display trait syndromes indicative of a resource-acquisitive strategy. This study contributes valuable data to global and national plant trait databases and advances theoretical understanding of the mechanisms underlying human-driven forest degradation in eastern China.

## 2. Results

### 2.1. Plant Morphological Traits

Morphological traits varied across plant species and differed between plants in disturbed and conserved forests (*p* < 0.05, permutational multivariate analysis of variance (PERMANOVA); Figure 1, Table 1 and Tables S2–S4). With the exception of bark thickness (BT) and bark tissue density (BTD), all other nine morphological traits showed statistically significant differences between the two forest types (*q* < 0.05, mixed-effects linear models with Benjamini–Hochberg false discovery rate (FDR) correction). Specifically, LT, LDMC, twig dry matter content (TDMC), twig tissue density (TTD), bark dry matter content (BDMC), stem dry matter content (SDMC), and stem tissue density (STD) were all significantly higher in disturbed forests than in conserved forests (*q* < 0.05). In contrast, leaf area (LA) and SLA were significantly greater in conserved forests relative to disturbed forests (*q* < 0.05; Figure 2, Tables S5 and S6).

Morphological traits of plants in disturbed forests exhibited coefficients of variation (CVs) ranging from 12.34% to 87.68% (mean = 35.31%), whereas those in conserved forests ranged from 12.45% to 89.73% (mean = 34.89%) (Table 1). Across both forest types, BT, LA, and SLA exhibited relatively high variation, whereas TDMC and LDMC showed comparatively low variation, as reflected by their respective CVs (Table 1). Notably, the CV values reported in Table 1 represent pooled individual-level variation across all sampled individuals; in contrast, paired-samples *t*-tests were conducted on species-level CV estimates for each morphological trait. These tests revealed that only TTD (*p* = 0.036, paired-samples *t*-tests) and BT (*p* = 0.049) showed a significantly higher CV in disturbed forests than in conserved forests. However, after correction for multiple comparisons using the FDR procedure, none of the morphological traits attained statistical significance at the adjusted threshold of *q* < 0.05. Moreover, under the prespecified equivalence bounds of 0.80–1.20, no trait met the criteria for equivalence as determined by Two One-sided Tests (TOST; Table S7). Given the current sample size and the defined equivalence margins, no systematic differences in the CVs of morphological traits were detected between disturbed and conserved forests.

### 2.2. Plant Ecological Stoichiometry Traits

Ecological stoichiometry traits varied across plant species and differed between plants in disturbed and conserved forests (*p* < 0.05, PERMANOVA; Figure 3, Table 2 and Tables S2–S4). Among the 24 ecological stoichiometry traits examined, 13 exhibited statistically significant differences between the two forest types (*q* < 0.05, mixed-effects linear models with FDR correction). Specifically, leaf carbon-to-nitrogen ratio (LCN), twig carbon content (TC), twig carbon-to-nitrogen ratio (TCN), bark carbon content (BC), bark carbon-to-phosphorus ratio (BCP), bark nitrogen-to-phosphorus ratio (BNP), stem carbon-to-phosphorus ratio (SCP), and stem nitrogen-to-phosphorus ratio (SNP) were significantly higher in disturbed forests than in conserved forests. Conversely, LN, leaf nitrogen-to-phosphorus ratio (LNP), twig nitrogen content (TN), bark phosphorus content (BP), and stem phosphorus content (SP) were significantly higher in conserved forests relative to disturbed forests (*q* < 0.05; Figure 4). In contrast, the remaining 11 ecological stoichiometry traits showed no statistically significant differences between plants from disturbed and conserved forests (*q* > 0.05; Figure 4).

Ecological stoichiometry traits of plants in disturbed forests exhibited CVs ranging from 2.03% to 47.62% (mean = 31.72%), whereas those in conserved forests ranged from 3.36% to 68.68% (mean = 32.34%) (Table 2). Across both forest types, TN, twig phosphorus content (TP), TCN, twig carbon-to-phosphorus ratio (TCP), twig nitrogen-to-phosphorus ratio (TNP), bark nitrogen content (BN), bark carbon-to-nitrogen ratio (BCN), BNP, stem nitrogen content (SN), SP, and stem carbon-to-nitrogen ratio (SCN) exhibited relatively high variation, whereas leaf carbon content (LC), TC, BC, and stem carbon content (SC) showed comparatively low variation, as indicated by their respective CVs (Table 2). Notably, the CV values reported in Table 2 represent pooled individual-level variation across all sampled individuals; in contrast, paired-samples *t*-tests were conducted on species-level CV estimates for each ecological stoichiometry trait. These tests indicated that only TC (*p* = 0.031, paired-samples *t*-tests) and TNP (*p* = 0.01) each showed significantly higher CVs in conserved forests than in disturbed forests. Yet, after FDR correction, none of the ecological stoichiometry traits reached statistical significance at *q* < 0.05. Likewise, under the same equivalence bounds (0.80–1.20), no trait satisfied the TOST equivalence criterion (Table S7). Thus, with the present sample size and specified equivalence margins, no systematic differences in the CVs of ecological stoichiometry traits were observed between disturbed and conserved forests.

## 3. Discussion

Fewer studies have investigated plant functional traits in anthropogenically disturbed vegetation compared with those in conserved vegetation [25,40,41,42]. Moreover, existing research on anthropogenic disturbances and plant functional traits has largely focused on herbaceous species affected by livestock grazing, fire, and biological invasion [43,44,45]. In contrast, the impacts of other widespread anthropogenic disturbances, such as timber harvesting, fuelwood collection, and livestock grazing, on plant functional traits in forest ecosystems remain comparatively understudied [12,25,46]. The present study comprehensively assessed 36 functional traits across leaves, twigs, bark, and stems of 23 common woody species in both anthropogenically disturbed and conserved subtropical forests in eastern China. This work helps bridge a critical knowledge gap in plant functional trait research, not only in subtropical China but also across diverse ecosystems worldwide.

Plant functional traits are jointly shaped by species-intrinsic attributes and external environmental conditions [47,48]. Environmental heterogeneity drives intraspecific trait variation and underpins the development of plant adaptive strategies, whereas interspecific trait variation primarily reflects phylogenetic divergence among species [49,50,51,52]. A persistent limitation of most previous studies is the variation in species composition across contrasting habitats, which impedes a clear separation of environmental effects on functional traits from confounding influences attributable to species identity. Some studies either overlook interspecific variation when investigating environment-driven trait shifts or rely on statistical approaches, such as two-way ANOVA, to partition species-environment interaction effects [53,54]. Departing from conventional research frameworks, this study focuses exclusively on plant species that co-occur in both disturbed and conserved subtropical forests, thereby eliminating confounding effects arising from interspecific turnover across habitats. This rigorous paired-species design, combined with mixed-effects linear models and paired-samples *t*-tests, enables a clean and robust assessment of how forest disturbance influences plant functional traits and their intraspecific variability. Our results indicate that forest disturbance reshapes plant functional strategies through intraspecific trait variation, rather than solely via community-level species turnover. By circumventing the pervasive methodological limitations of prior comparative studies, this approach provides robust, intraspecific evidence for plant functional adaptation to forest disturbance.

Plant functional traits serve as robust proxies for characterizing plant life history strategies [55,56]. Disturbed forests are subject to periodic or persistent external perturbations that destabilize microhabitats, drive fluctuations in resource availability, and intensify interspecific competition. In contrast, conserved forests maintain stable microclimate conditions, sustain soil nutrient supply, and exert relatively low competitive pressure, thereby establishing fundamentally contrasting habitat regimes for plant growth and development [38,57]. Previous studies consistently report that plants in disturbed forests exhibit lower SLA, higher tissue dry matter content and density (in bark, stems, twigs, and leaves), reduced N and P concentrations, and elevated C concentrations in these tissues. Collectively, these trait shifts reflect a resource-conservative strategy adapted to chronic environmental stress. By contrast, plants in stable, conserved forests display opposing trait syndromes and adopt a resource-acquisitive strategy [4,25,58]. We explicitly tested our stated hypotheses against the empirical findings and found only partial support. Although several traits in disturbed forests shifted toward the predicted conservative strategy, multiple stoichiometry traits showed no statistically significant differences between forest types: LC, SC, BN, SN, LP, and TP did not differ significantly between disturbed and conserved forests. These mixed results indicate that the resource-conservative syndrome induced by disturbance is not uniformly expressed across all plant organs and that plant trait responses to disturbance are organ-specific. The contrasting morphological and stoichiometry trait syndromes observed between the two forest types arise from multiple non-exclusive environmental drivers. First, disturbed forests exhibit reduced canopy cover (55% at the disturbed forest functional trait sampling site versus 75% at the conserved forest site; these canopy estimates reflect unreplicated site characterizations rather than replicated quantitative measurements). This reduction increases light availability in the understory. Concurrently, lower LA and SLA—together with higher tissue density and enhanced mechanical robustness of plant organs—align with canonical sun-leaf characteristics: a well-documented adaptive strategy to high-light environments that emphasizes mechanical stability and light use efficiency over rapid biomass accumulation under open, high irradiance conditions. Although we recognize that a sun-leaf morphological response to canopy opening is conceptually distinct from a resource-conservative strategy—and that our current experimental design cannot disentangle these two potential drivers—we report these trait shifts as consistent with elevated light exposure. Second, consistent with prior regional studies [38,57], disturbed forest habitats are characterized by limited soil nutrient availability. The soil nutrient data presented here comprise unreplicated, site-level measurements collected from plots adjacent to our function trait sampling locations; they are provided solely as descriptive contextual background and are not intended to support causal inference. Specifically, surface (0–10 cm) soil concentrations of organic carbon (C) and total nitrogen (N) were markedly lower in the disturbed forest plot (21.47 and 1.93 mg g^−1^, respectively) than in the conserved forest plot (49.75 and 3.82 mg g^−1^, respectively). In contrast, the difference in total phosphorus (P) was comparatively modest (0.36 vs. 0.40 mg g^−1^). Given the small magnitude of this disparity—and the similarly small difference observed in total P concentration overall—it is unlikely to explain the considerably larger differences in P concentrations detected in bark and stems. Plant available P, not total P, represents the ecologically relevant metric for assessing soil P supply to plants. Accordingly, these soil data are treated exclusively as contextual background information and are not interpreted as direct evidence linking soil nutrient status to plant tissue elemental composition. Notably, departing from conventional analytical approaches, which frequently neglect species turnover across forest types, our analysis, restricted to overlapping species in disturbed and conserved forests, revealed a distinctive pattern: functional trait variation in disturbed forests did not decline relative to that in conserved forests. Consequently, our hypothesis—that forest disturbance reduces functional trait variation—was not supported. These findings provide novel insight into how plant functional traits respond to forest disturbances, suggesting that environmental instability may sustain, or even amplify, functional diversity within species, rather than uniformly constraining it.

Beyond the analysis of plant life history strategy differentiation between disturbed and conserved forests, the trait database compiled for overlapping species in this study holds substantial potential to advance ecological research. The field-measured functional traits presented herein can be used to characterize shifts in the functional composition of forest communities and to track dynamic changes in functional diversity following anthropogenic disturbances [59]. Furthermore, they enable a holistic assessment of how forest disturbances reshape both abiotic and biotic environmental conditions and reconfigure ecosystem-level functional performance [60]. Such intraspecific trait records also provide robust empirical evidence to disentangle disturbance-driven ecological responses, thereby enhancing the scientific rigor of ecological assessments and strengthening conservation initiatives in subtropical forests [4,10].

Nevertheless, this study has several limitations. First, trait sampling was conducted at a single site per forest type, thereby confounding disturbance treatment with site-specific effects. Although our overlapping species paired comparisons provide valuable insights into intraspecific trait responses, the influence of disturbance cannot be fully disentangled from inherent differences among sites. Second, the study cannot fully exclude the possibility that disturbance has pre-filtered the local species pool: species with resource-acquisitive strategies may have been selectively eliminated from disturbed forests, leaving only those with resource-conservative strategies to overlap across the two forest types. If such a filtering process occurred, the observed trait differences between forest types may reflect, at least in part, pre-selection from the regional species pool rather than purely intraspecific trait variation in response to disturbance. Third, the study focused exclusively on morphological and ecological stoichiometry traits of nutritive organs across 23 common species in two forest types; functional traits beyond those measured, including root, reproductive, and physiological traits, as well as traits of understory shrubs, lianas, and herbaceous species, were not considered. Fourth, the study employed a static field sampling design, which precludes detection of temporal shifts in plant functional traits and life history strategies under prolonged or cumulative disturbances. Future research should integrate community species surveys, broaden the taxonomic scope, and extend the disturbance gradient. Critically, such efforts must combine long-term, site-based monitoring with molecular, physiological, and demographic approaches. These integrative efforts will advance our mechanistic understanding of how disturbances drive variation in plant functional traits and shape divergent ecological strategies. Ultimately, this refined knowledge base will provide a more robust theoretical foundation for conserving forest biodiversity and guiding ecologically informed restoration of disturbed forest ecosystems.

## 4. Materials and Methods

### 4.1. Study Area

Located in Suichang County, Zhejiang Province, in eastern China, Jiulong Mountain National Nature Reserve covers a geographic extent bounded by 118°49′–118°55′ E longitude and 28°19′–28°24′ N latitude. Stretching 8.8 km from east to west and 10.5 km from north to south, the reserve encompasses a total area of 55.25 km^2^ (Figure 5). As a critical ecological transition zone connecting Zhejiang, Fujian, and Jiangxi provinces, it is designated both globally and nationally as a priority site for biodiversity conservation—attributable to its unique transitional geographical position and exceptionally rich biological resources [61,62]. The region experiences a mid-subtropical humid monsoon climate, characterized by high atmospheric humidity and abundant annual precipitation. Meteorological records indicate a mean annual temperature of 16.2 °C, with extreme temperatures ranging from −10.5 °C to 37.0 °C. Annual precipitation averages 1856 mm, mean relative humidity remains at 80%, and the area receives approximately 1925 h of sunshine per year [10]. Geologically and pedologically, the terrain and substrate are predominantly derived from weathered acidic igneous rocks. Mountain red-yellow soil is the dominant soil type throughout the reserve, while paddy soil occurs only sporadically in low-lying topographic depressions [25]. Owing to its relatively isolated location and limited transportation infrastructure, the reserve retains extensive tracts of primary subtropical evergreen broad-leaved forest—a habitat increasingly rare in eastern China and a principal focus of conservation efforts. Additional conservation priorities include numerous rare and endangered animal and plant species [4,10].

### 4.2. History of Sampling Sites and Vegetation Survey

Field sampling was conducted in the Jiulong Mountain National Nature Reserve and its surrounding protective buffer zone—a region anticipated to be incorporated into the reserve in the near future—both areas exhibiting contrasting histories of anthropogenic disturbance (Figure 5). Prior to 1983, both areas experienced comparable intensity and types of anthropogenic disturbance. In 1983, however, the reserve was designated as a provincial-level nature reserve and subsequently elevated to national status in 2003. Since 1983, the reserve’s management authority has enforced stringent regulations prohibiting all forms of anthropogenic disturbances. In contrast, the surrounding protective buffer zone—though subject to fire suppression—has continued to experience intermittent, intermediate-intensity anthropogenic disturbances, including timber harvesting, fuelwood collection, and livestock grazing [4].

Preliminary vegetation surveys were conducted across 66 standardized 20 m × 20 m plots to characterize plant community composition and guide the targeted selection of overlapping species for functional trait sampling. These plot-based surveys served exclusively for species screening and baseline community characterization—not for in situ individual-level trait measurements. A total of 22 plots were established within the nature reserve to represent well-conserved forest stands, while 44 plots were deployed in the surrounding protective buffer zone to represent forests experiencing ongoing anthropogenic disturbance. Plot placement encompassed all dominant forest types in the region. For each plot, basic environmental variables—including GPS coordinates, elevation, slope gradient, slope aspect, and bedrock coverage—were recorded. Within every plot, all woody individuals with a diameter at breast height (DBH) ≥ 5 cm were taxonomically identified; DBH, tree height, and crown dimensions were also measured and documented.

### 4.3. Plant Functional Trait Assessment

Based on field plot inventory data, 23 plant species occurring in both disturbed and conserved forests were selected for functional trait assessment (Table 3). During functional trait sampling, individual plants were selected across conserved and disturbed forest zones at comparable elevations; inclusion within pre-established community plots was not a requirement. Within each forest type, five healthy, mature, and representative individuals per species were chosen for sampling, with similar DBH to ensure comparability. From each selected individual, canopy branches were collected from sun-exposed portions of the canopy using a retractable high-branch pruner; four branches were harvested from distinct canopy positions per plant. From each harvested branch, five intact, fully expanded broad leaves were collected; for coniferous taxa, ten mature needle leaves were substituted. Additionally, one 20 cm long twig, aged 2–3 years, was collected. This sampling protocol yielded 20–40 mature leaves and four twig samples per individual plant. Furthermore, three bark samples and three stem samples were collected at breast height from each target plant, using a sickle and an increment borer. Notably, stem samples were not collected for *Phyllostachys edulis*, owing to fundamental anatomical and structural differences between bamboo culms and woody tree stems. Similarly, bark samples were omitted for *Eurya muricata*, *Itea omeiensis*, *Phyllostachys edulis*, and *Rhododendron ovatum*, either because these species lack a distinct bark layer altogether or because their bark is exceptionally thin, fragile, or tightly adherent to the underlying xylem, thereby precluding the reliable collection of intact, representative samples (Table S1). All collected samples were immediately placed into plastic zip-lock bags upon field collection and stored in a portable cooler to maintain sample integrity for subsequent laboratory testing and analyses.

Morphological traits were quantified following standardized protocols [63,64]. LT was measured using a vernier caliper with a precision of 0.01 mm. For each measurement, five intact leaves were stacked and measured collectively, avoiding the primary veins. Gentle, consistent manual pressure was applied to the caliper jaws to prevent leaf compression. The mean thickness across these collected leaves was recorded as the individual-level LT. The area of all sampled leaves was scanned and analyzed using the WinFOLIA leaf area analysis system (Regent Instruments Inc., Quebec, QC, Canada); average single leaf area was calculated by dividing the total scanned area by the number of leaves. Leaf fresh mass was determined using an electronic balance with a precision of 0.001 g. Subsequently, leaf samples were oven-dried at 80 °C for 48 h until constant mass was achieved, enabling accurate determination of leaf dry mass. Leaf volume was estimated as the product of mean LA and LT. From these primary measurements, several key leaf morphological traits were derived: SLA was defined as LA divided by leaf dry mass, and LDMC was expressed as the ratio of leaf dry mass to fresh mass. Similarly, BT was determined by measuring three bark samples per individual plant using a vernier caliper (Guanglu Measuring Instrument Co., Ltd., Guilin, China); the mean value was used as the individual-level BT. Fresh masses of twig, bark, and stem tissues were measured using a high-precision electronic balance, and tissue volumes were determined via the water displacement method. All above measurements were completed on the day of sample collection. Twig, bark, and stem samples were oven-dried at 80 °C for 72 h until constant mass to obtain dry mass values. TDMC, BDMC, and SDMC were calculated as the respective ratios of dry mass to fresh mass. TTD, BTD, and STD were computed as dry mass divided by the corresponding tissue volume.

After morphological trait measurements, all plant tissue samples were homogenized into a fine, uniform powder and sieved through a 0.2 mm mesh prior to chemical elemental analysis. Total C and N concentrations in each tissue type were quantified using a Vario MACRO Cube elemental analyzer (Elementar Analysensysteme GmbH, Langenselbold, Germany). Specifically, these comprised LC and LN; TC and TN; BC and BN; and SC and SN. Total P concentrations, namely LP, TP, BP, and SP, were determined by inductively coupled plasma optical emission spectrometry (ICP-OES) on an iCAP 6300 spectrometer (Thermo Fisher Scientific, Cambridge, UK). Finally, key stoichiometric ratios (carbon-to-nitrogen ratio, carbon-to-phosphorus ratio, and nitrogen-to-phosphorus ratio) were calculated for all four tissue types, yielding the following stoichiometric parameters: leaf (LCN, LCP, and LNP), twig (TCN, TCP, and TNP), bark (BCN, BCP, and BNP), and stem (SCN, SCP, and SNP).

Several prior studies have investigated forest community assembly and functional trait variation within the same reserve. The functional trait datasets employed in those studies exhibit both overlapping and independent relationships with those used in the present study. Guan et al. [25] established the most comprehensive functional trait dataset for this region and serves as the foundational data source for functional trait analyses in subsequent local studies—including those of Liu et al. [4,10] and the present study. Specifically, the morphological trait data utilized in Liu et al. [4] represent only a small subset of the trait records compiled by Guan et al. [25] and adopted in the present study. In contrast, the functional trait data used in Liu et al. [10] are independent of those employed in the present study, although they also originate from the original dataset reported by Guan et al. [25].

### 4.4. Data Analysis

All statistical analyses were performed in R (version 4.1.3) [65]. LT, LA, and SLA of *Cryptomeria japonica* var. *sinensis*, *Cunninghamia lanceolata*, and *Pinus massoniana*—for which LT and LA were measured using widely adopted yet methodologically inappropriate protocols for conifers (WinFOLIA calculates projected needle area, which does not represent true one-sided leaf area and is therefore unsuitable for SLA computation; furthermore, LT values derived from this approach are not directly comparable across coniferous and broad-leaved species), thereby undermining the reliability of the derived SLA values. Additionally, stem trait data for *P. edulis* and bark trait data for *E. muricata*, *I. omeiensis*, *P. edulis*, and *R. ovatum* were excluded from all statistical analyses. However, these four leaf trait data for the three coniferous species are provided in Supplementary Table S1 solely for reference—to facilitate cross-study comparisons with research that employs identical measurement protocols. Functional trait variability was quantified using the CV, calculated as the ratio of the standard deviation to the corresponding trait mean.

Principal component analysis (PCA) was conducted to visualize the multivariate distribution of functional traits across the 23 overlapping plant species in disturbed and conserved forests. To quantify overall differences in functional trait composition between the two forest types, PERMANOVA was performed using standardized Euclidean distances (vegan::adonis2). To preserve the paired experimental design—where each of the 23 target species was sampled in both disturbed and conserved forests—permutation constraints were applied at the species level: during each permutation iteration, forest type labels were randomly swapped only between the disturbed and conserved counterparts of the same species. A total of 9999 permutations were used for the PERMANOVA test to ensure statistical robustness. The primary analysis includes all 23 species-forest type combinations. For any given trait, if data are entirely missing for a specific species × forest type combination, we first compute the trait centroid—defined as the mean value across all individuals within that combination. Centroids that remain missing after this step are imputed using the overall mean of all available centroids for that trait. This centroid-level imputation is applied exclusively to the PCA and PERMANOVA; raw individual-level data are left unimputed. To evaluate the robustness of our findings to this imputation approach, we additionally performed a complete-case sensitivity analysis.

Mixed-effects linear models were employed to assess statistically significant differences in functional traits between forest types. To address right skewness and heteroscedasticity in the functional trait data, natural logarithmic transformations were applied prior to model fitting. Forest type was specified as a fixed effect, with conserved forests serving as the reference category and disturbed forests as the comparator. Plant species was included as a random effect to account for non-independence among individuals of the same species. Standardized log(DBH) was included as a covariate to control for the influence of stem diameter on functional traits. For each functional trait, we first compared two candidate random-effects structures using maximum likelihood (ML) estimation: (i) a random-intercept model (RI): log(trait) ~ forest + log(DBH) + (1|species), and (ii) a random-intercept–random-slope model (RI+RS): log(trait) ~ forest + log(DBH) + (1 + forest|species). The RI+RS model was selected only if it yielded both a lower AIC and a statistically significant improvement in fit (approximate likelihood ratio test, *p* < 0.05). The final model for each trait—using the selected random-effects structure—was refitted using restricted maximum likelihood (REML), and fixed-effect estimates and associated inferential statistics were extracted from this REML-fitted model. Denominator degrees of freedom for fixed-effect tests were approximated conservatively at the species clustering level: df = effective number of species—1. When data for all 23 species were available for a given trait, df = 22; when one or more species lacked data for that trait, df was reduced accordingly.

Paired-samples *t*-tests were used to compare the CVs of functional traits between forest types. For each trait, the CV was calculated within each “species × forest type” combination, based on measurements from five individuals per combination. Species served as the unit of pairing: for each species, its CVs computed separately in disturbed and conserved forests formed a matched pair. For traits lacking complete data on five individuals in either forest type, only species with valid CV estimates in both forest types were retained for pairing; consequently, the number of effective pairs varied across traits—specifically, 19, 20, or 23 pairs. Prior to statistical testing, CV values were log-transformed. Paired-sample *t*-tests were then conducted on the log-transformed CVs, with all resulting *p* values adjusted for multiple comparisons using the FDR procedure (stats::p.adjust(method=“BH”)). Equivalence testing was performed on the log-ratio scale using the TOST procedure. The prespecified equivalence margin of 0.80–1.20 was defined for the untransformed CV ratio, representing the smallest ecologically meaningful difference [66].

## 5. Conclusions

Overall, we examined the functional traits of overlapping plant species across anthropogenically disturbed and conserved forest habitats. Our results revealed that individuals in disturbed forests exhibited significantly higher values of LT, LDMC, TDMC, TTD, BDMC, BTD, SDMC, and STD, a series of traits collectively indicative of a resource-conservative strategy. This strategy was further corroborated by lower N concentrations in leaves and twigs and lower P concentrations in bark and stems. In contrast, plants in conserved forests displayed opposing trait syndromes—consistent with a resource-acquisitive strategy. Notably, however, analyses restricted to overlapping species revealed no evidence of reduced functional trait variability in disturbed forests relative to conserved forests. Our study thus advances a more nuanced understanding of how anthropogenic disturbances shape plant functional traits and associated trait-based life history strategies in forests of eastern China.

## Figures and Tables

**Figure 1:**
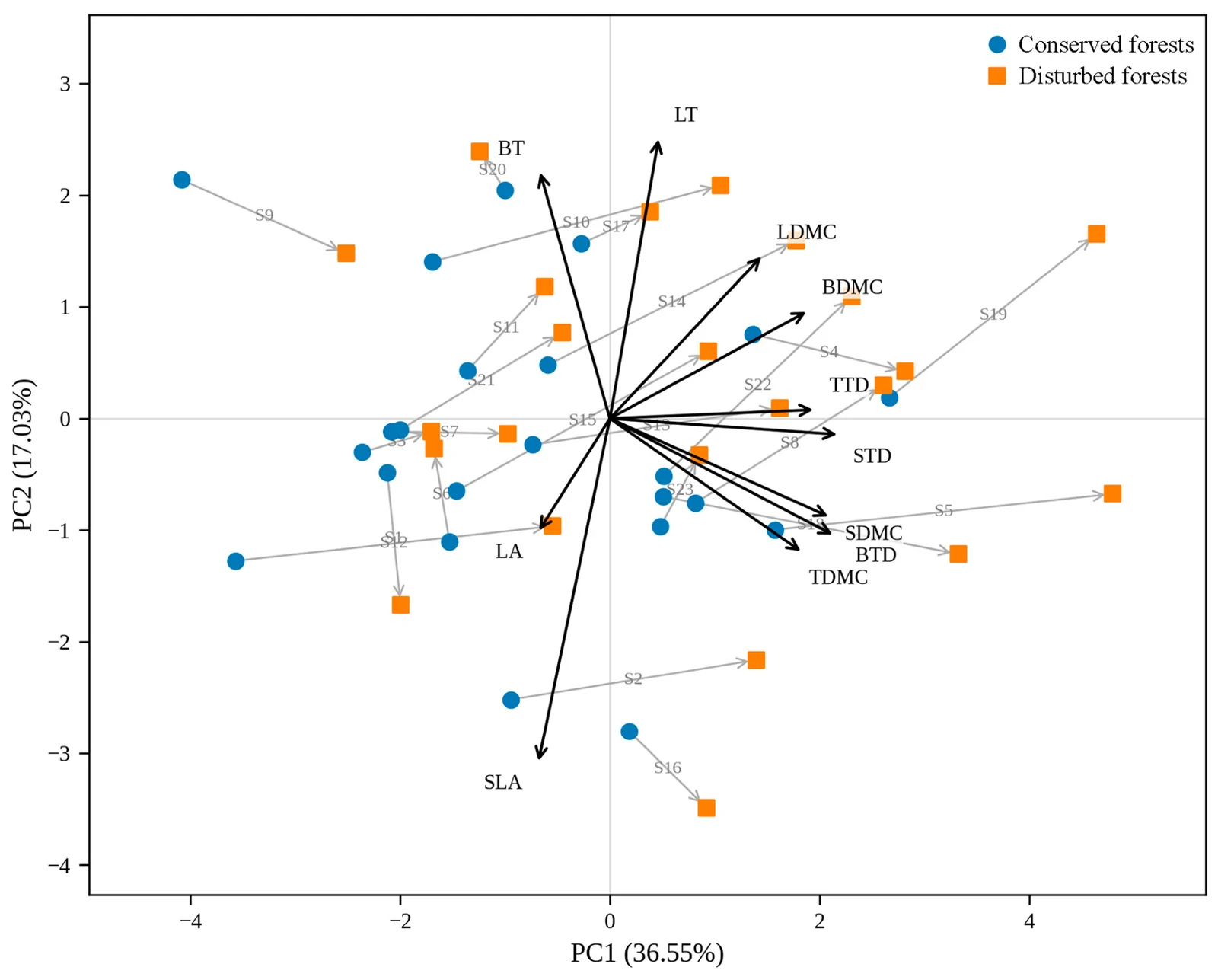
Principal component analysis (PCA) biplot of plant morphological traits, illustrating paired species observations from disturbed and conserved forests in eastern China. LT, leaf thickness; LA, leaf area; SLA, specific leaf area; LDMC, leaf dry matter content; TDMC, twig dry matter content; TTD, twig tissue density; BT, bark thickness; BDMC, bark dry matter content; BTD, bark tissue density; SDMC, stem dry matter content; STD, stem tissue density. S1, *Acer davidii*; S2, *Acer elegantulum*; S3, *Alniphyllum fortunei*; S4, *Castanopsis eyrei*; S5, *Celtis biondii*; S6, *Choerospondias axillaris*; S7, *Cryptomeria japonica* var. *sinensis*; S8, *Cornus hongkongensis*; S9, *Cunninghamia lanceolata*; S10, *Eurya muricata*; S11, *Itea omeiensis*; S12, *Lindera erythrocarpa*; S13, *Loropetalum chinense*; S14, *Machilus thunbergii*; S15, *Phoebe sheareri*; S16, *Phyllostachys edulis*; S17, *Pinus massoniana*; S18, *Prunus schneideriana*; S19, *Quercus glauca*; S20, *Rhododendron latoucheae*; S21, *Rhododendron ovatum*; S22, *Schima superba;* S23, *Toxicodendron succedaneum*.

**Figure 2:**
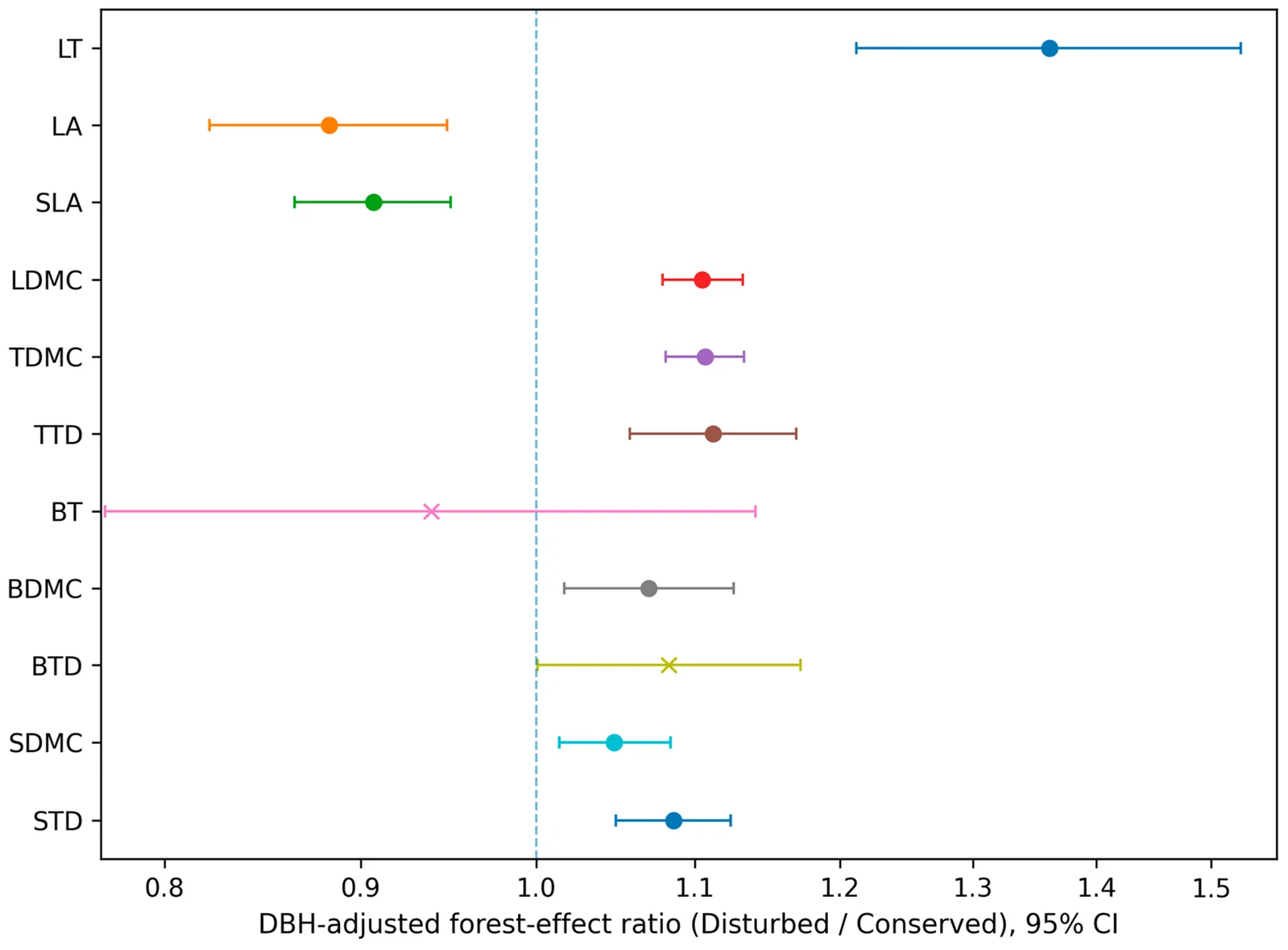
Diameter at breast height (DBH)-adjusted forest-type effect ratios (disturbed vs. conserved forests) and their 95% confidence intervals, estimated from mixed-effects linear models fitted to plant morphological traits. LT, leaf thickness; LA, leaf area; SLA, specific leaf area; LDMC, leaf dry matter content; TDMC, twig dry matter content; TTD, twig tissue density; BT, bark thickness; BDMC, bark dry matter content; BTD, bark tissue density; SDMC, stem dry matter content; STD, stem tissue density.

**Figure 3:**
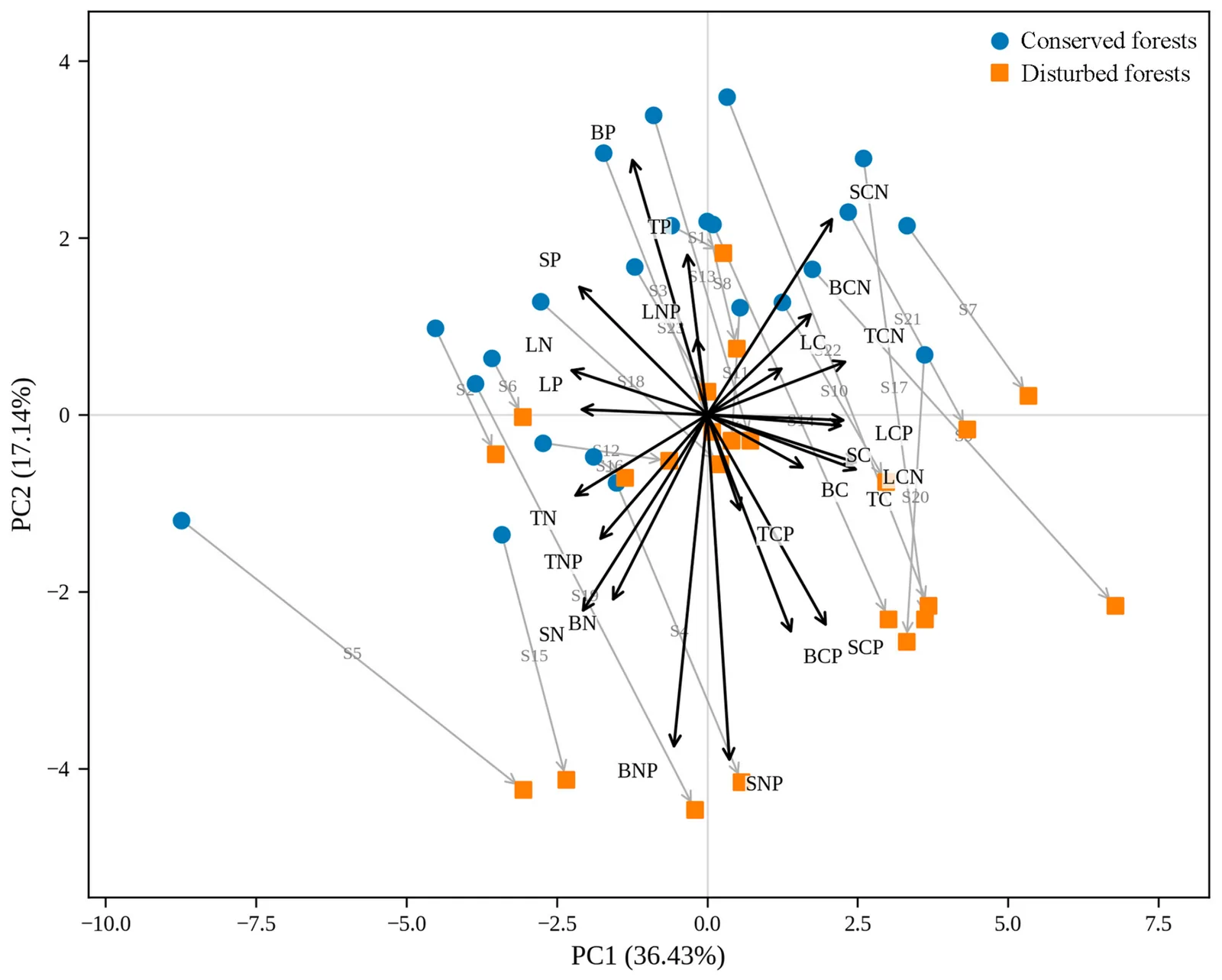
Principal component analysis (PCA) biplot of plant ecological stoichiometry traits, illustrating paired species observations from disturbed and conserved forests in eastern China. LC, leaf carbon content; LN, leaf nitrogen content; LP, leaf phosphorus content; LCN, leaf carbon-to-nitrogen ratio; LCP, leaf carbon-to-phosphorus ratio; LNP, leaf nitrogen-to-phosphorus ratio; TC, twig carbon content; TN, twig nitrogen content; TP, twig phosphorus content; TCN, twig carbon-to-nitrogen ratio; TCP, twig carbon-to-phosphorus ratio; TNP, twig nitrogen-to-phosphorus ratio; BC, bark carbon content; BN, bark nitrogen content; BP, bark phosphorus content; BCN, bark carbon-to-nitrogen ratio; BCP, bark carbon-to-phosphorus ratio; BNP, bark nitrogen-to-phosphorus ratio; SC, stem carbon content; SN, stem nitrogen content; SP, stem phosphorus content; SCN, stem carbon-to-nitrogen ratio; SCP, stem carbon-to-phosphorus ratio; SNP, stem nitrogen-to-phosphorus ratio. S1, *Acer davidii*; S2, *Acer elegantulum*; S3, *Alniphyllum fortunei*; S4, *Castanopsis eyrei*; S5, *Celtis biondii*; S6, *Choerospondias axillaris*; S7, *Cryptomeria japonica* var. *sinensis*; S8, *Cornus hongkongensis*; S9, *Cunninghamia lanceolata*; S10, *Eurya muricata*; S11, *Itea omeiensis*; S12, *Lindera erythrocarpa*; S13, *Loropetalum chinense*; S14, *Machilus thunbergii*; S15, *Phoebe sheareri*; S16, *Phyllostachys edulis*; S17, *Pinus massoniana*; S18, *Prunus schneideriana*; S19, *Quercus glauca*; S20, *Rhododendron latoucheae*; S21, *Rhododendron ovatum*; S22, *Schima superba;* S23, *Toxicodendron succedaneum*.

**Figure 4:**
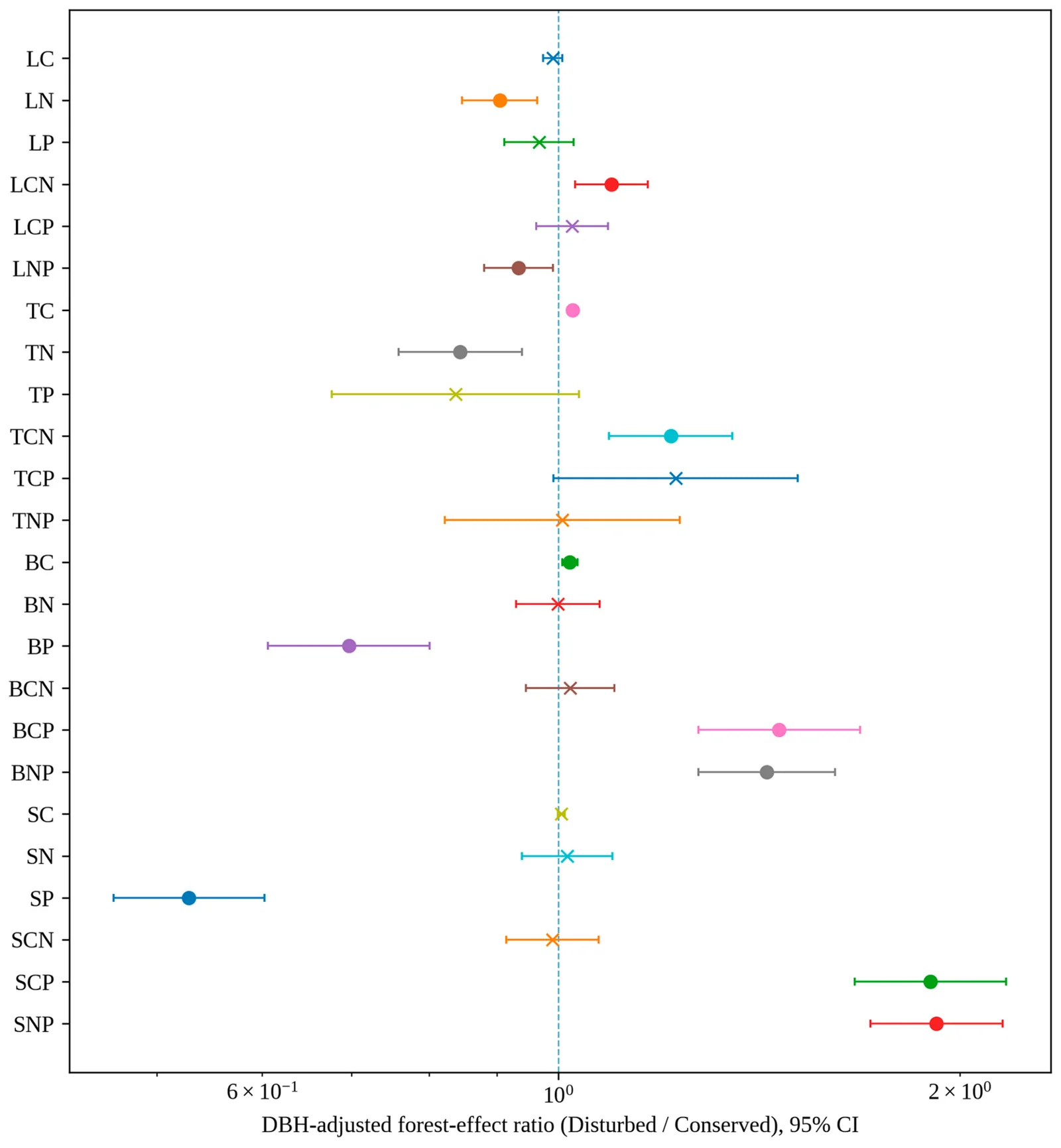
DBH-adjusted forest-type effect ratios (disturbed vs. conserved forests) and their 95% confidence intervals, estimated from mixed-effects linear models fitted to plant ecological stoichiometry traits. LC, leaf carbon content; LN, leaf nitrogen content; LP, leaf phosphorus content; LCN, leaf carbon-to-nitrogen ratio; LCP, leaf carbon-to-phosphorus ratio; LNP, leaf nitrogen-to-phosphorus ratio; TC, twig carbon content; TN, twig nitrogen content; TP, twig phosphorus content; TCN, twig carbon-to-nitrogen ratio; TCP, twig carbon-to-phosphorus ratio; TNP, twig nitrogen-to-phosphorus ratio; BC, bark carbon content; BN, bark nitrogen content; BP, bark phosphorus content; BCN, bark carbon-to-nitrogen ratio; BCP, bark carbon-to-phosphorus ratio; BNP, bark nitrogen-to-phosphorus ratio; SC, stem carbon content; SN, stem nitrogen content; SP, stem phosphorus content; SCN, stem carbon-to-nitrogen ratio; SCP, stem carbon-to-phosphorus ratio; SNP, stem nitrogen-to-phosphorus ratio.

**Figure 5:**
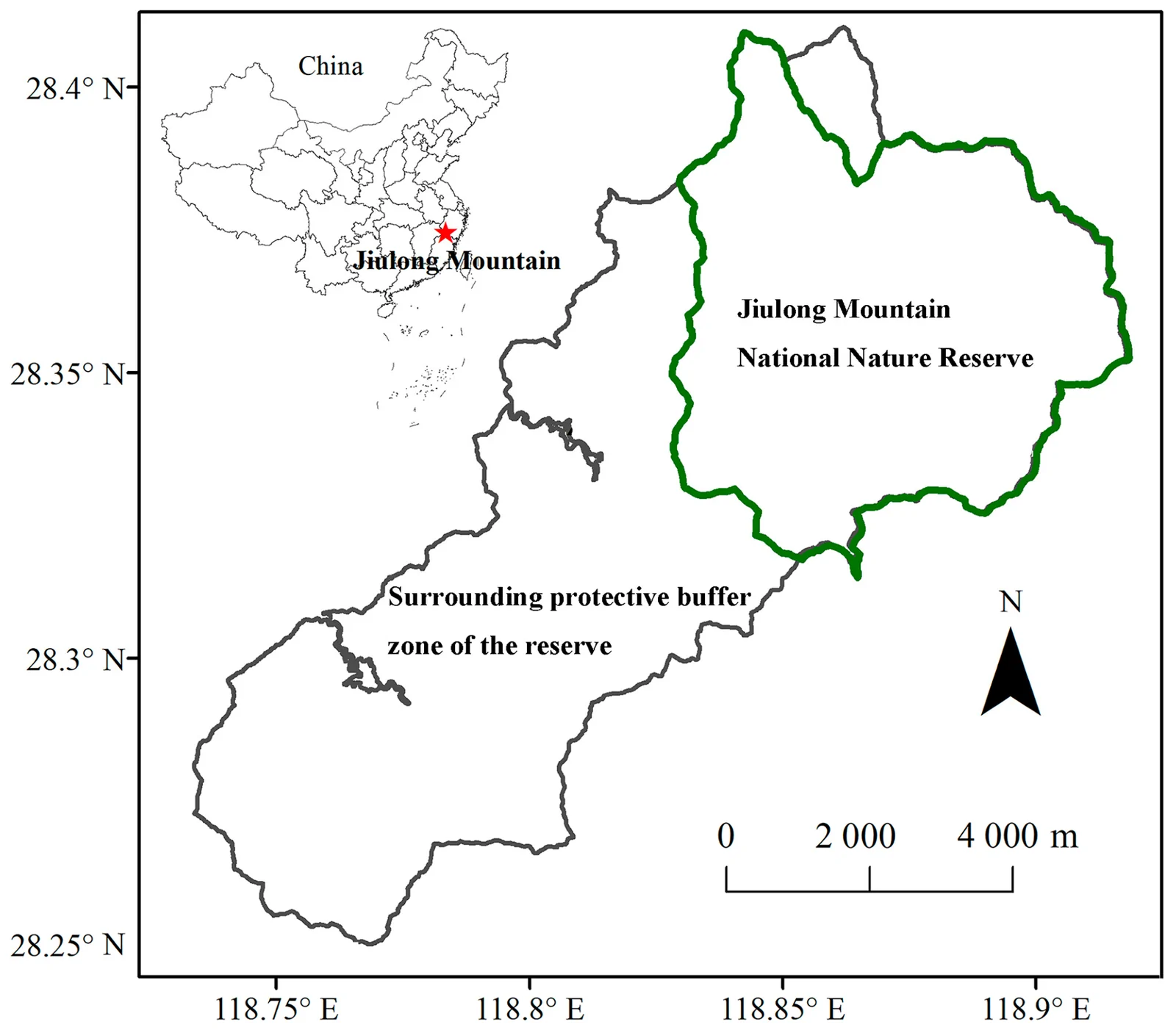
Location of the Jiulong Mountain National Nature Reserve and its surrounding protective buffer zone in eastern China.

**Table 1:** Plant morphological traits in disturbed and conserved forests in eastern China.

Morphological Traits	Disturbed Forests					Conserved Forests				
	N	Mean	Max	Min	CV (%)	N	Mean	Max	Min	CV (%)
LT (mm)	100	0.40 ± 0.12	0.78	0.10	31.01	100	0.29 ± 0.11	0.90	0.13	38.44
LA (cm^2^)	100	20.62 ± 10.60	54.78	3.39	51.41	100	23.93 ± 14.22	81.59	4.11	59.43
SLA (cm^2^ g^−1^)	100	125.95 ± 54.85	285.47	57.16	43.55	100	137.78 ± 55.72	308.70	59.60	40.44
LDMC (g g^−1^)	115	0.42 ± 0.06	0.57	0.31	13.18	115	0.38 ± 0.05	0.52	0.26	13.82
TDMC (g g^−1^)	115	0.53 ± 0.07	0.69	0.39	12.34	115	0.48 ± 0.06	0.64	0.33	12.45
TTD (g cm^−3^)	115	0.54 ± 0.10	0.78	0.24	18.83	115	0.49 ± 0.10	0.73	0.25	20.08
BT (mm)	95	4.03 ± 3.53	19.22	0.84	87.68	95	4.31 ± 3.86	21.32	0.71	89.73
BDMC (g g^−1^)	95	0.55 ± 0.10	0.83	0.33	18.91	95	0.51 ± 0.08	0.84	0.36	16.39
BTD (g cm^−3^)	95	0.51 ± 0.17	0.96	0.16	33.39	95	0.46 ± 0.12	0.69	0.20	25.13
SDMC (g g^−1^)	110	0.54 ± 0.11	0.73	0.28	21.08	110	0.51 ± 0.09	0.64	0.25	17.59
STD (g cm^−3^)	110	0.56 ± 0.15	1.28	0.28	26.44	110	0.51 ± 0.11	0.77	0.25	21.85

LT, leaf thickness; LA, leaf area; SLA, specific leaf area; LDMC, leaf dry matter content; TDMC, twig dry matter content; TTD, twig tissue density; BT, bark thickness; BDMC, bark dry matter content; BTD, bark tissue density; SDMC, stem dry matter content; STD, stem tissue density.

**Table 2:** Plant ecological stoichiometry traits in disturbed and conserved forests in eastern China.

Ecological Stoichiometry Traits	Disturbed Forests					Conserved Forests				
	N	Mean	Max	Min	CV (%)	N	Mean	Max	Min	CV (%)
LC (mg g^−1^)	115	460.34 ± 26.18	556.79	396.87	5.69	115	464.73 ± 29.06	512.86	375.89	6.25
LN (mg g^−1^)	115	13.68 ± 4.34	25.11	5.75	31.75	115	14.99 ± 4.25	26.90	7.55	28.37
LP (mg g^−1^)	115	0.93 ± 0.29	1.68	0.53	31.46	115	0.97 ± 0.31	2.07	0.46	32.31
LCN	115	37.38 ± 13.06	84.44	18.64	34.94	115	33.72 ± 10.42	64.08	15.78	30.90
LCP	115	543.06 ± 169.17	908.41	264.20	31.15	115	536.34 ± 191.02	1032.60	237.11	35.62
LNP	115	14.84 ± 2.47	19.67	9.41	16.66	115	15.88 ± 2.67	28.85	10.24	16.81
TC (mg g^−1^)	115	468.22 ± 9.72	492.05	447.00	2.08	115	456.65 ± 15.37	482.07	413.81	3.36
TN (mg g^−1^)	115	3.11 ± 1.33	9.51	1.08	42.87	115	3.66 ± 1.46	8.76	1.00	39.81
TP (mg g^−1^)	115	0.48 ± 0.19	1.15	0.17	39.85	115	0.60 ± 0.32	2.75	0.13	53.04
TCN	115	179.80 ± 81.40	446.85	49.27	45.27	115	149.20 ± 74.02	476.41	48.28	49.61
TCP	115	1123.97 ± 441.62	2786.70	399.24	39.29	115	960.49 ± 507.01	3499.27	166.67	52.79
TNP	115	6.81 ± 2.67	22.30	2.03	39.24	115	7.58 ± 5.20	29.00	1.52	68.68
BC (mg g^−1^)	95	463.64 ± 35.14	530.61	382.61	7.58	95	454.27 ± 33.69	516.61	376.23	7.42
BN (mg g^−1^)	95	5.50 ± 2.13	12.63	2.09	38.81	95	5.48 ± 2.13	11.83	1.69	38.86
BP (mg g^−1^)	95	0.45 ± 0.13	0.78	0.19	29.02	95	0.63 ± 0.11	0.96	0.38	17.44
BCN	95	98.88 ± 45.03	248.69	32.33	45.54	95	95.88 ± 41.24	298.48	33.51	43.01
BCP	95	1141.83 ± 448.60	2778.02	601.42	39.29	95	747.60 ± 155.57	1216.41	477.68	20.81
BNP	95	12.51 ± 4.53	28.11	5.70	36.20	95	8.87 ± 3.61	26.44	2.81	40.69
SC (mg g^−1^)	110	467.14 ± 9.41	490.55	447.52	2.01	110	464.82 ± 15.90	568.19	385.79	3.42
SN (mg g^−1^)	110	2.00 ± 0.84	4.47	0.78	42.01	110	1.97 ± 0.92	5.83	0.78	46.57
SP (mg g^−1^)	110	0.26 ± 0.12	0.73	0.11	47.19	110	0.48 ± 0.19	1.35	0.28	39.30
SCN	110	276.56 ± 110.37	604.53	101.68	39.91	110	278.93 ± 105.65	590.41	66.17	37.88
SCP	110	2105.27 ± 776.23	4249.02	624.32	36.87	110	1076.14 ± 313.52	1752.35	338.90	29.13
SNP	110	8.12 ± 2.98	19.96	2.85	36.67	110	4.18 ± 1.43	9.15	1.82	34.17

LC, leaf carbon content; LN, leaf nitrogen content; LP, leaf phosphorus content; LCN, leaf carbon-to-nitrogen ratio; LCP, leaf carbon-to-phosphorus ratio; LNP, leaf nitrogen-to-phosphorus ratio; TC, twig carbon content; TN, twig nitrogen content; TP, twig phosphorus content; TCN, twig carbon-to-nitrogen ratio; TCP, twig carbon-to-phosphorus ratio; TNP, twig nitrogen-to-phosphorus ratio; BC, bark carbon content; BN, bark nitrogen content; BP, bark phosphorus content; BCN, bark carbon-to-nitrogen ratio; BCP, bark carbon-to-phosphorus ratio; BNP, bark nitrogen-to-phosphorus ratio; SC, stem carbon content; SN, stem nitrogen content; SP, stem phosphorus content; SCN, stem carbon-to-nitrogen ratio; SCP, stem carbon-to-phosphorus ratio; SNP, stem nitrogen-to-phosphorus ratio.

**Table 3:** Basic information on 23 overlapping plant species in disturbed and conserved forests of eastern China.

Species	Genus	Family	Growth Form	Life Form
*Acer davidii*	*Acer*	Sapindaceae	Tree	Deciduous
*Acer elegantulum*	*Acer*	Sapindaceae	Tree	Deciduous
*Alniphyllum fortunei*	*Alniphyllum*	Styracaceae	Tree	Deciduous
*Castanopsis eyrei*	*Castanopsis*	Fagaceae	Tree	Evergreen
*Celtis biondii*	*Celtis*	Cannabaceae	Tree	Deciduous
*Choerospondias axillaris*	*Choerospondias*	Anacardiaceae	Tree	Deciduous
*Cryptomeria japonica* var. *sinensis*	*Cryptomeria*	Cupressaceae	Tree	Evergreen
*Cornus hongkongensis*	*Cornus*	Cornaceae	Tree	Evergreen
*Cunninghamia lanceolata*	*Cunninghamia*	Cupressaceae	Tree	Evergreen
*Eurya muricata*	*Eurya*	Pentaphylacaceae	Shrub	Evergreen
*Itea omeiensis*	*Itea*	Iteaceae	Shrub	Evergreen
*Lindera erythrocarpa*	*Lindera*	Lauraceae	Tree	Deciduous
*Loropetalum chinense*	*Loropetalum*	Hamamelidaceae	Shrub	Evergreen
*Machilus thunbergii*	*Machilus*	Lauraceae	Tree	Evergreen
*Phoebe sheareri*	*Phoebe*	Lauraceae	Tree	Evergreen
*Phyllostachys edulis*	*Phyllostachys*	Poaceae	Arborescent bamboo species	Evergreen
*Pinus massoniana*	*Pinus*	Pinaceae	Tree	Evergreen
*Prunus schneideriana*	*Prunus*	Rosaceae	Tree	Deciduous
*Quercus glauca*	*Quercus*	Fagaceae	Tree	Evergreen
*Rhododendron latoucheae*	*Rhododendron*	Ericaceae	Shrub	Evergreen
*Rhododendron ovatum*	*Rhododendron*	Ericaceae	Shrub	Evergreen
*Schima superba*	*Schima*	Theaceae	Tree	Evergreen
*Toxicodendron succedaneum*	*Toxicodendron*	Anacardiaceae	Tree	Deciduous

## Data Availability

The original contributions presented in this study are included in the article/Supplementary Materials. Further inquiries can be directed to the corresponding author.

## Supplementary Materials

The following supporting information can be downloaded at https://www.mdpi.com/article/10.3390/plants15192962/s1, Table S1: Individual-level morphological and ecological stoichiometry traits of overlapping plant species in disturbed and conserved forests in eastern China. Table S2: Eigenvalues and the proportion of variance explained by principal components from principal component analysis of plant functional traits. Table S3: Trait loadings for principal components 1 and 2 from principal component analysis of plant functional traits. Table S4: Results of permutational multivariate analysis of variance (PERMANOVA) for plant functional traits, including primary paired analyses based on imputed centroids and complete-case sensitivity analyses. Table S5: Mixed-linear model results examining the effects of forest type on plant functional traits, including regression coefficients, test statistics, FDR-adjusted *p* values, and R^2^ values. Table S6: Model comparisons between random-intercept (RI) and random-intercept–random-slope (RI—RS) mixed-effects linear models for plant functional traits. Table S7: Paired-samples *t*-tests and Two One-Sided Tests (TOSTs) for equivalence of the trait coefficient of variation, with Benjamini–Hochberg false discovery rate (FDR) correction.
