# Supplementary material for: Plant Trait-Based Life Strategies of Overlapping Species Differ Between Anthropogenically Disturbed and Conserved Forests
Source: Plants (Basel). 2026 Sep 28;15(19):2962. doi: 10.3390/plants15192962 (PMC13666656; doi:10.3390/plants15192962)
Supplement: Supplementary file 1 [file plants-15-02962-s001.zip › Table S2-S7.pdf]

Table S2 Eigenvalues and the proportion of variance explained by principal components from principal component analysis of plant functional traits.

Trait groups	PC	Eigenvalue	Explained variance	Cumulative
Morphological traits	PC1	4.295	35.80%	35.80%
	PC2	2.152	17.93%	53.73%
	PC3	1.674	13.95%	67.68%
	PC4	1.086	9.05%	76.73%
	PC5	0.939	7.82%	84.55%
Ecological stoichiometry traits	PC1	8.462	35.26%	35.26%
	PC2	4.182	17.42%	52.68%
	PC3	2.693	11.22%	63.90%
	PC4	2.118	8.83%	72.73%
	PC5	1.586	6.61%	79.34%

Table S3 Trait loadings for principal components 1 and 2 from principal component analysis of plant functional traits. LT, leaf thickness; LA, leaf area; SLA, specific leaf area; LDMC, leaf dry matter content; LTD, leaf tissue density; TDMC, twig dry matter content; TTD, twig tissue density; BT, bark thickness; BDMC, bark dry matter content; BTD, bark tissue density; SDMC, stem dry matter content; STD, stem tissue density; LC, leaf carbon content; LN, leaf nitrogen content; LP, leaf phosphorus content; LCN, leaf carbon-to-nitrogen ratio; LCP, leaf carbon-to-phosphorus ratio; LNP, leaf nitrogen-to-phosphorus ratio; TC, twig carbon content; TN, twig nitrogen content; TP, twig phosphorus content; TCN, twig carbon-to-nitrogen ratio; TCP, twig carbon-to-phosphorus ratio; TNP, twig nitrogen-to-phosphorus ratio; BC, bark carbon content; BN, bark nitrogen content; BP, bark phosphorus content; BCN, bark carbon-to-nitrogen ratio; BCP, bark carbon-to-phosphorus ratio; BNP, bark nitrogen-to-phosphorus ratio; SC, stem carbon content; SN, stem nitrogen content; SP, stem phosphorus content; SCN, stem carbon-to-nitrogen ratio; SCP, stem carbon-to-phosphorus ratio; SNP, stem nitrogen-to-phosphorus ratio.

Functional traits	PC1 loading	PC2 loading
LT	0.155	-0.336
LA	-0.331	0.341
SLA	-0.107	0.759
LDMC	0.365	-0.670
LTD	0.172	-0.434
TDMC	0.806	0.148
TTD	0.808	-0.223
BT	-0.519	-0.572
BDMC	0.530	-0.401
BTD	0.796	0.153
SDMC	0.856	0.306
STD	0.877	0.180
LC	0.421	-0.178
LN	-0.760	0.002
LP	-0.703	0.130
LCN	0.838	0.031

LCP	0.759	-0.127
LNP	-0.069	-0.293
TC	0.845	0.151
TN	-0.758	0.134
TP	-0.157	-0.551
TCN	0.780	-0.069
TCP	0.236	0.397
TNP	-0.577	0.392
BC	0.567	0.150
BN	-0.532	0.371
BP	-0.446	-0.680
BCN	0.555	-0.137
BCP	0.487	0.581
BNP	-0.188	0.776
SC	0.707	0.023
SN	-0.648	0.512
SP	-0.683	-0.345
SCN	0.664	-0.531
SCP	0.629	0.579
SNP	0.122	0.889

Table S4 Results of permutational multivariate analysis of variance (PERMANOVA) for plant functional traits, including primary paired analyses based on imputed centroids and complete-case sensitivity analyses.

**Trait groups**	**Analysis**	**Species pairs**	**Pseudo-F**	**R^2**	**Permutation p**
Morphological traits	Primary (paired, imputed centroids)	23	4.378	0.090	< 0.001
	Complete-case sensitivity	16	3.323	0.100	< 0.001
Ecological stoichiometry traits	Primary (paired, imputed centroids)	23	6.703	0.132	< 0.001
	Complete-case sensitivity	19	5.166	0.125	< 0.001

Table S5 Mixed-linear model results examining the effects of forest type on plant functional traits, including regression coefficients, test statistics, FDR-adjusted p-values, and R^2 values. LT, leaf thickness; LA, leaf area; SLA, specific leaf area; LDMC, leaf dry matter content; LTD, leaf tissue density; TDMC, twig dry matter content; TTD, twig tissue density; BT, bark thickness; BDMC, bark dry matter content; BTD, bark tissue density; SDMC, stem dry matter content; STD, stem tissue density; LC, leaf carbon content; LN, leaf nitrogen content; LP, leaf phosphorus content; LCN, leaf carbon-to-nitrogen ratio; LCP, leaf carbon-to-phosphorus ratio; LNP, leaf nitrogen-to-phosphorus ratio; TC, twig carbon content; TN, twig nitrogen content; TP, twig phosphorus content; TCN, twig carbon-to-nitrogen ratio; TCP, twig carbon-to-phosphorus ratio; TNP, twig nitrogen-to-phosphorus ratio; BC, bark carbon content; BN, bark nitrogen content; BP,

bark phosphorus content; BCN, bark carbon-to-nitrogen ratio; BCP, bark carbon-to-phosphorus ratio; BNP, bark nitrogen-to-phosphorus ratio; SC, stem carbon content; SN, stem nitrogen content; SP, stem phosphorus content; SCN, stem carbon-to-nitrogen ratio; SCP, stem carbon-to-phosphorus ratio; SNP, stem nitrogen-to-phosphorus ratio.

Functional traits	Model	N	β±SE	t (df)	D/C ratio [95% CI]	p	FDR q	R^2m	R^2c
LT	RI+RS	200	0.310±0.055	5.64 (22)	1.363 [1.216, 1.528]	< 0.001	< 0.001	0.155	0.831
LA	RI	200	-0.123±0.034	-3.65 (22)	0.884 [0.824, 0.948]	0.001	0.003	0.011	0.838
SLA	RI	200	-0.098±0.022	-4.39 (22)	0.907 [0.866, 0.950]	< 0.001	< 0.001	0.015	0.843
LDMC	RI	230	0.099±0.011	8.64 (22)	1.104 [1.078, 1.131]	< 0.001	< 0.001	0.118	0.638
LTD	RI+RS	200	-0.212±0.053	-4.00 (22)	0.809 [0.725, 0.903]	< 0.001	0.002	0.096	0.736
TDMC	RI	230	0.102±0.011	9.04 (22)	1.107 [1.082, 1.133]	< 0.001	< 0.001	0.138	0.613
TTD	RI+RS	230	0.107±0.024	4.44 (22)	1.113 [1.059, 1.170]	< 0.001	< 0.001	0.064	0.732
BT	RI+RS	190	-0.071±0.094	-0.75 (22)	0.932 [0.767, 1.132]	0.459	0.533	0.002	0.745
BDMC	RI+RS	190	0.068±0.024	2.80 (22)	1.070 [1.018, 1.126]	0.010	0.018	0.035	0.750
BTD	RI+RS	190	0.080±0.038	2.13 (22)	1.083 [1.002, 1.171]	0.044	0.067	0.015	0.866
SDMC	RI+RS	230	0.057±0.021	2.67 (22)	1.059 [1.013, 1.107]	0.014	0.023	0.016	0.751
STD	RI+RS	230	0.097±0.024	3.97 (22)	1.101 [1.047, 1.158]	< 0.001	0.002	0.032	0.807
LC	RI+RS	230	-0.009±0.008	-1.14 (22)	0.991 [0.975, 1.007]	0.267	0.339	0.005	0.873
LN	RI+RS	230	-0.101±0.031	-3.24 (22)	0.904 [0.847, 0.964]	0.004	0.007	0.026	0.837
LP	RI+RS	230	-0.032±0.029	-1.12 (22)	0.968 [0.912, 1.028]	0.273	0.339	0.003	0.835
LCN	RI+RS	230	0.092±0.030	3.05 (22)	1.097 [1.030, 1.168]	0.006	0.011	0.020	0.839
LCP	RI+RS	230	0.023±0.030	0.78 (22)	1.023 [0.962, 1.089]	0.445	0.533	0.001	0.836
LNP	RI+RS	230	-0.069±0.029	-2.42 (22)	0.933 [0.880, 0.990]	0.024	0.038	0.040	0.576
TC	RI+RS	230	0.025±0.004	5.72 (22)	1.026 [1.016, 1.035]	< 0.001	<0.001	0.165	0.912
TN	RI+RS	230	-0.168±0.051	-3.27 (22)	0.845 [0.760, 0.941]	0.004	0.007	0.038	0.657
TP	RI+RS	230	-0.174±0.103	-1.70 (22)	0.840 [0.679, 1.040]	0.104	0.139	0.039	0.614
TCN	RI+RS	230	0.193±0.051	3.76 (22)	1.213 [1.090, 1.350]	0.001	0.003	0.047	0.681
TCP	RI+RS	230	0.200±0.102	1.97 (22)	1.221 [0.989, 1.507]	0.062	0.086	0.051	0.620
TNP	RI+RS	230	0.007±0.098	0.07 (22)	1.007 [0.822, 1.233]	0.948	0.975	0.000	0.692
BC	RI+RS	190	0.020±0.006	3.26 (22)	1.020 [1.007, 1.034]	0.004	0.007	0.017	0.912
BN	RI+RS	190	0.000±0.034	0.01 (22)	1.000 [0.932, 1.073]	0.994	0.994	0.000	0.857
BP	RI+RS	190	-0.361±0.066	-5.43 (22)	0.697 [0.607, 0.800]	< 0.001	< 0.001	0.333	0.754
BCN	RI+RS	190	0.020±0.036	0.55 (22)	1.020 [0.946, 1.100]	0.586	0.659	0.001	0.872
BCP	RI+RS	190	0.381±0.066	5.74 (22)	1.464 [1.276, 1.680]	< 0.001	< 0.001	0.305	0.777
BNP	RI+RS	190	0.361±0.056	6.42 (22)	1.435 [1.277, 1.613]	< 0.001	< 0.001	0.204	0.871
SC	RI	230	0.005±0.003	2.04 (22)	1.005 [1.000, 1.011]	0.053	0.077	0.009	0.489
SN	RI	230	0.013±0.036	0.37 (22)	1.014 [0.940, 1.092]	0.713	0.778	0.000	0.543
SP	RI+RS	230	-0.650±0.061	-10.70 (22)	0.522 [0.460, 0.592]	< 0.001	< 0.001	0.440	0.690
SCN	RI	230	-0.008±0.037	-0.22 (22)	0.992 [0.919, 1.071]	0.828	0.877	0.000	0.547
SCP	RI+RS	230	0.655±0.061	10.70 (22)	1.926 [1.696, 2.186]	< 0.001	< 0.001	0.429	0.694
SNP	RI+RS	230	0.663±0.053	12.40 (22)	1.941 [1.738, 2.169]	< 0.001	< 0.001	0.491	0.801

Table S6 Model comparisons between random-intercept (RI) and random-intercept–random-slope

(RI–RS) mixed-effects linear models for plant functional traits. LT, leaf thickness; LA, leaf area; SLA, specific leaf area; LDMC, leaf dry matter content; LTD, leaf tissue density; TDMC, twig dry matter content; TTD, twig tissue density; BT, bark thickness; BDMC, bark dry matter content; BTD, bark tissue density; SDMC, stem dry matter content; STD, stem tissue density; LC, leaf carbon content; LN, leaf nitrogen content; LP, leaf phosphorus content; LCN, leaf carbon-to-nitrogen ratio; LCP, leaf carbon-to-phosphorus ratio; LNP, leaf nitrogen-to-phosphorus ratio; TC, twig carbon content; TN, twig nitrogen content; TP, twig phosphorus content; TCN, twig carbon-to-nitrogen ratio; TCP, twig carbon-to-phosphorus ratio; TNP, twig nitrogen-to-phosphorus ratio; BC, bark carbon content; BN, bark nitrogen content; BP, bark phosphorus content; BCN, bark carbon-to-nitrogen ratio; BCP, bark carbon-to-phosphorus ratio; BNP, bark nitrogen-to-phosphorus ratio; SC, stem carbon content; SN, stem nitrogen content; SP, stem phosphorus content; SCN, stem carbon-to-nitrogen ratio; SCP, stem carbon-to-phosphorus ratio; SNP, stem nitrogen-to-phosphorus ratio.

**Functional traits**	**Selected model**	**ΔAIC (RS−RI)**	**LRT**	**LRT p**
LT	RI+RS	-36.75	40.75	< 0.001
LA	RI	1.94	2.06	0.358
SLA	RI	2.77	1.23	0.541
LDMC	RI	1.61	2.39	0.303
LTD	RI+RS	-23.80	27.80	< 0.001
TDMC	RI	0.86	3.14	0.208
TTD	RI+RS	-8.70	12.70	0.002
BT	RI+RS	-8.44	12.44	0.002
BDMC	RI+RS	-13.62	17.62	< 0.001
BTD	RI+RS	-29.28	33.28	< 0.001
SDMC	RI+RS	-5.71	9.71	0.008
STD	RI+RS	-8.77	12.77	0.002
LC	RI+RS	-61.10	65.10	< 0.001
LN	RI+RS	-18.21	22.21	< 0.001
LP	RI+RS	-8.68	12.68	0.002
LCN	RI+RS	-12.50	16.50	< 0.001
LCP	RI+RS	-8.66	12.66	0.002
LNP	RI+RS	-19.55	23.55	< 0.001
TC	RI+RS	-122.53	126.53	< 0.001
TN	RI+RS	-5.26	9.26	0.010
TP	RI+RS	-67.06	71.06	< 0.001
TCN	RI+RS	-4.84	8.84	0.012
TCP	RI+RS	-63.81	67.81	< 0.001
TNP	RI+RS	-67.60	71.60	< 0.001
BC	RI+RS	-11.77	15.77	< 0.001
BN	RI+RS	-4.46	8.46	0.015
BP	RI+RS	-68.54	72.54	< 0.001
BCN	RI+RS	-6.63	10.63	0.005
BCP	RI+RS	-58.23	62.23	< 0.001
BNP	RI+RS	-46.36	50.36	< 0.001

SC	RI	0.39	3.61	0.164
SN	RI	2.97	1.03	0.597
SP	RI+RS	-10.26	14.26	< 0.001
SCN	RI	2.95	1.05	0.593
SCP	RI+RS	-9.70	13.70	0.001
SNP	RI+RS	-19.67	23.67	< 0.001

Table S7 Paired-samples t-tests and Two One-sided Tests (TOST) for equivalence of the trait coefficient of variation, with Benjamini–Hochberg false discovery rate (FDR) correction. LT, leaf thickness; LA, leaf area; SLA, specific leaf area; LDMC, leaf dry matter content; LTD, leaf tissue density; TDMC, twig dry matter content; TTD, twig tissue density; BT, bark thickness; BDMC, bark dry matter content; BTD, bark tissue density; SDMC, stem dry matter content; STD, stem tissue density; LC, leaf carbon content; LN, leaf nitrogen content; LP, leaf phosphorus content; LCN, leaf carbon-to-nitrogen ratio; LCP, leaf carbon-to-phosphorus ratio; LNP, leaf nitrogen-to-phosphorus ratio; TC, twig carbon content; TN, twig nitrogen content; TP, twig phosphorus content; TCN, twig carbon-to-nitrogen ratio; TCP, twig carbon-to-phosphorus ratio; TNP, twig nitrogen-to-phosphorus ratio; BC, bark carbon content; BN, bark nitrogen content; BP, bark phosphorus content; BCN, bark carbon-to-nitrogen ratio; BCP, bark carbon-to-phosphorus ratio; BNP, bark nitrogen-to-phosphorus ratio; SC, stem carbon content; SN, stem nitrogen content; SP, stem phosphorus content; SCN, stem carbon-to-nitrogen ratio; SCP, stem carbon-to-phosphorus ratio; SNP, stem nitrogen-to-phosphorus ratio.

**Functional traits**	**N**	**CV ratio [90% CI]**	**t (df)**	**p**	**FDR q**	**TOST p**	**Equivalent**	**MDE**
LT	20	0.878 [0.723, 1.066]	-1.16 (19)	0.261	0.522	0.210	No	39.4%
LA	20	0.915 [0.712, 1.177]	-0.61 (19)	0.549	0.732	0.183	No	53.6%
SLA	20	1.082 [0.836, 1.400]	0.53 (19)	0.603	0.738	0.248	No	55.3%
LDMC	23	0.822 [0.621, 1.087]	-1.21 (22)	0.241	0.522	0.436	No	61.2%
LTD	20	0.880 [0.664, 1.166]	-0.79 (19)	0.442	0.636	0.283	No	61.8%
TDMC	23	0.845 [0.666, 1.073]	-1.21 (22)	0.238	0.522	0.348	No	50.2%
TTD	23	0.702 [0.535, 0.922]	-2.23 (22)	0.036	0.336	0.790	No	59.1%
BT	19	1.329 [1.055, 1.673]	2.14 (18)	0.047	0.336	0.773	No	48.3%
BDMC	19	1.299 [0.966, 1.747]	1.53 (18)	0.143	0.469	0.676	No	65.9%
BTD	19	1.018 [0.784, 1.321]	0.12 (18)	0.908	0.945	0.144	No	56.1%
SDMC	23	1.152 [0.868, 1.529]	0.86 (22)	0.400	0.636	0.404	No	62.2%
STD	23	1.131 [0.886, 1.442]	0.87 (22)	0.395	0.636	0.339	No	51.5%
LC	23	1.486 [1.090, 2.025]	2.20 (22)	0.039	0.336	0.876	No	69.7%
LN	23	1.067 [0.857, 1.328]	0.51 (22)	0.615	0.738	0.183	No	45.3%
LP	23	0.957 [0.759, 1.206]	-0.33 (22)	0.747	0.840	0.099	No	48.5%
LCN	23	1.098 [0.872, 1.382]	0.69 (22)	0.495	0.685	0.256	No	48.2%
LCP	23	1.028 [0.832, 1.270]	0.23 (22)	0.824	0.899	0.111	No	43.4%
LNP	23	0.923 [0.723, 1.178]	-0.56 (22)	0.579	0.738	0.162	No	51.6%
TC	23	0.700 [0.532, 0.921]	-2.23 (22)	0.036	0.336	0.793	No	59.8%
TN	23	0.832 [0.645, 1.074]	-1.24 (22)	0.229	0.522	0.397	No	54.5%
TP	23	0.807 [0.640, 1.017]	-1.59 (22)	0.126	0.469	0.474	No	48.5%

TCN	23	0.849 [0.678, 1.062]	-1.26 (22)	0.222	0.522	0.328	No	46.7%
TCP	23	0.816 [0.665, 1.002]	-1.70 (22)	0.104	0.466	0.434	No	42.0%
TNP	23	0.626 [0.475, 0.825]	-2.92 (22)	0.008	0.287	0.930	No	60.1%
BC	19	1.071 [0.788, 1.456]	0.39 (18)	0.704	0.818	0.264	No	69.0%
BN	19	1.311 [1.001, 1.716]	1.74 (18)	0.099	0.466	0.711	No	58.5%
BP	19	1.277 [0.888, 1.836]	1.17 (18)	0.258	0.522	0.615	No	86.0%
BCN	19	1.348 [1.028, 1.769]	1.91 (18)	0.072	0.435	0.767	No	59.0%
BCP	19	1.223 [0.867, 1.725]	1.02 (18)	0.323	0.598	0.538	No	79.9%
BNP	19	1.232 [0.973, 1.558]	1.53 (18)	0.142	0.469	0.575	No	49.5%
SC	23	0.973 [0.613, 1.543]	-0.10 (22)	0.918	0.945	0.238	No	119.8%
SN	23	1.139 [0.909, 1.426]	0.99 (22)	0.332	0.598	0.346	No	46.8%
SP	23	1.145 [0.855, 1.532]	0.80 (22)	0.435	0.636	0.392	No	64.5%
SCN	23	1.183 [0.957, 1.463]	1.36 (22)	0.188	0.522	0.454	No	43.7%
SCP	23	1.136 [0.863, 1.496]	0.80 (22)	0.434	0.636	0.368	No	59.9%
SNP	23	0.997 [0.801, 1.242]	-0.02 (22)	0.984	0.984	0.081	No	45.5%
